# Preoperative Inflammatory Control and Surgical Outcome of Vitrectomy in Intermediate Uveitis

**DOI:** 10.1155/2017/5946240

**Published:** 2017-03-30

**Authors:** Yong Un Shin, Joo Young Shin, Dae Joong Ma, Heeyoon Cho, Hyeong Gon Yu

**Affiliations:** ^1^Department of Ophthalmology, Hanyang University College of Medicine, Seoul, Republic of Korea; ^2^Department of Ophthalmology, Seoul National University Hospital Healthcare System Gangnam Center, Seoul, Republic of Korea; ^3^Department of Ophthalmology, Seoul National University College of Medicine, Seoul, Republic of Korea

## Abstract

*Purpose*. To demonstrate the long-term effectiveness of vitrectomy for intermediate uveitis (IU) and to determine whether complete control of inflammation before vitrectomy is necessary. *Methods*. This retrospective study included 66 eyes of 66 patients with IU who underwent vitrectomy for vitreoretinal complications. Eyes were followed for at least 12 months after vitrectomy. The degree of inflammation control and visual acuity were compared before and after vitrectomy. These parameters were compared according to the presence of complete inflammation control before surgery. *Results*. The indications of vitrectomy included epiretinal membrane (26 eyes), vitreous opacity (21 eyes), and tractional retinal detachment (12 eyes), among others. Uveitic attacks did not occur in 44 of the 66 patients after vitrectomy. The numbers of uveitis attacks, local steroid injections, and systemic medications significantly decreased, and vision meaningfully improved after vitrectomy. These parameters did not differ significantly, regardless of the presence of preoperative inflammation. *Conclusions*. This study showed that vitrectomy is a good modality to manage vitreoretinal complications associated with IU, even if complete control of intraocular inflammation is not achieved before vitrectomy.

## 1. Introduction

Intermediate uveitis (IU) is a subset of uveitis wherein the vitreous is the major site of inflammation [1]. Typical ophthalmologic findings of IU include yellow-white inflammatory aggregates typically found in the inferior vitreous (snowballs) and exudates on the inferior pars plana (snowbanks) [2]. The clinical course of IU varies considerably, ranging from a self-limiting process to a chronic, refractory course characterized by multiple exacerbations and remissions [3].

Pars plana vitrectomy is normally indicated in IU when there are secondary vitreoretinal complications such as an epiretinal membrane, tractional retinal detachment, rhegmatogenous retinal detachment, vitreous opacity, macular edema, and vitreous hemorrhage or when intraocular inflammation has not subsided despite aggressive medical treatment [2, 4]. Several authors have reported that vitrectomy effectively alleviated intraocular inflammation and improved visual acuity in IU [5–9]. To achieve optimal outcomes, it was recommended that a clinically quiescent state with no intraocular inflammation should be maintained for at least three months before surgery [10–14]. However, several authors have reported successful results without complete control of inflammation prior to surgery for various subtypes of uveitis [15–17].

In the present study, we aimed to demonstrate the long-term effectiveness of vitrectomy in IU patients with accompanying vitreoretinal complications. In particular, we investigated the control of intraocular inflammation as well as visual outcomes and asked if preoperative control of intraocular inflammation affected long-term visual outcomes.

## 2. Materials and Methods

We identified patients with IU who had undergone pars plana vitrectomy between October 1995 and December 2010 at the Seoul National University Hospital and had a minimum follow-up of 12 months. The clinical diagnosis of IU was made based on the criteria proposed by the SUN Working Group, when the vitreous is the major site of inflammation and may be associated with peripheral vascular sheathing and macular edema [1]. The typical ophthalmic indicators of IU were inflammation of the vitreous, snowballs, and/or snowbanks, as observed by indirect ophthalmoscopy. In patients with bilateral IU who underwent vitrectomy in both eyes, we included the eye with the more severe clinical course. We excluded patients less than 20 years old or patients with IU-associated rhegmatogenous retinal detachment. This retrospective study was approved by the Institutional Review Board of the Seoul National University Hospital and adhered to the tenets of the Declaration of Helsinki.

At their first visit, patients underwent thorough ophthalmic examinations including best-corrected visual acuity (BCVA) and intraocular pressure measurement, slit-lamp biomicroscopy, and fundus examination. All patients also underwent comprehensive laboratory tests to exclude uveitis associated with systemic diseases such as syphilis, sarcoidosis, or tuberculosis.

Patients were initially treated with periocular steroid injection and/or oral administration of steroids and/or nonsteroidal anti-inflammatory drugs, based on the treating physicians' discretion. In addition, immunomodulatory drugs such as cyclosporine, mycophenolate mofetil, or azathioprine were added in cases where intraocular inflammation was not well controlled.

Vitrectomy was performed for inflammation-related vitreoretinal complications including epiretinal membranes, vitreous opacities, tractional retinal detachment, macular edema, or vitreous hemorrhage by two experienced surgeons. Despite systemic and periocular anti-inflammatory treatment, not all patients showed complete control of intraocular inflammation at the time of surgery. If active inflammation was observed at the time of surgery, the dosage of systemic steroids was increased. Phacoemulsification and secondary intraocular lens implantation were indicated in patients with more than a moderate-grade cataract. Encircling or segmental buckling was also performed in case of tractional retinal detachment, if it was judged to be necessary or helpful by the surgeon.

The patients were followed up 1 day, 1 week, and 4 weeks after surgery, after which the follow-up period was lengthened. BCVA, intraocular pressure, and ocular inflammation status were documented. Patients with postoperative recurrence of intraocular inflammation were managed with periocular and oral steroids and/or nonsteroidal anti-inflammatory drugs and/or immunomodulatory drugs at the treating physicians' discretion.

The primary goals of the present study were to examine postoperative uveitis recurrence (defined as a vitreous haze of grade 1 or more) and to compare the degree of pre- and postvitrectomy intraocular inflammation control and BCVA. The degree of inflammation control was evaluated by the average number of uveitis attacks per year, the average number of local steroid injections per year, the degree of vitreous haze, and the average number of systemic medications used. The BCVA was measured using a Snellen visual acuity chart and converted to the logarithm of the minimal angle of resolution (logMAR) for analysis. The secondary goal of this study was to determine whether or not complete preoperative inflammation control had influenced postoperative inflammatory control and visual outcome. Patients were classified into two groups based on the presence or absence of active inflammation at the time of vitrectomy. The absence of active inflammation was defined as grade 0.5+ or less cells in the anterior chamber and 0.5+ or less haze in the vitreous with no involvement of the posterior segment [1, 18].

We compared the rates of postoperative uveitis attacks, the degrees of postoperative inflammation control, and the BCVA at the last follow-up between the two groups. Any complications associated with vitrectomy were also identified. We used an independent *t*-test, paired *t*-test, Pearson's chi-square test, and Pearson's correlation test to analyze the demographic data and outcome measures. Statistical analyses were performed using SPSS for Windows (ver. 18.0, Statistical Package for the Social Sciences, SPSS Inc., Chicago, IL); *P* values < 0.05 were considered significant.

## 3. Results

We included a total of 66 eyes from 66 patients (40 males and 26 females) with a mean age of 48.76 ± 13.51. The average period from IU onset to vitrectomy was 32.63 ± 40.95 months. Demographic data are summarized in Table 1.

Vitrectomy was performed for any of the various vitreoretinal complications secondary to IU, including epiretinal membranes, vitreous opacities, tractional retinal detachment, macular edema, and vitreous hemorrhage (Table 1). Various gauges (20, 23, and 25) were utilized in the vitrectomy procedure. Thirty-six eyes (54.5%) received the 23- or 25-gauge vitrectomy, and the others (30 eyes, 45.5%) received the 20-gauge vitrectomy. Phacoemulsification and secondary intraocular lens implantation were performed on 31 of 66 eyes (47.0%), encircling or buckling was performed in six of 66 eyes (9.1%), and lensectomy was performed in three of 66 eyes (4.5%).

After vitrectomy, the patients were followed for a mean of 48.91 ± 40.38 months. There were no uveitis attacks in 44 patients (66.7%) after vitrectomy. 22 patients (33.3%) experienced one or more of such attacks during the postoperative period; 11 patients (50.0%) experienced one event, four patients (18.2%) experienced two events, and seven patients (31.8%) experienced three or more events. The first uveitis attack arose within six months of vitrectomy in 17 patients (77.3%). The average number of attacks decreased from 1.43 ± 0.89 times per year in the preoperative period to 0.23 ± 0.49 times per year in the postoperative period (*P* < 0.001), and the average number of local steroid injections decreased from 0.80 ± 1.04 injections per year to 0.10 ± 0.42 (*P* < 0.001). The grade of vitreous haze decreased from 0.59 ± 0.78 before surgery to 0.06 ± 0.24 at the last follow-up (*P* < 0.001). There were only four patients with grade 1 vitreous haze or more at the last follow-up, compared with 30 patients with a vitreous haze of grade 1 or more at the preoperative examination. The average number of systemic medications decreased from 0.79 ± 1.03 three months before vitrectomy to 0.38 ± 0.67 at the last follow-up (*P* = 0.001). Forty-eight of 66 patients (72.7%) did not receive any medical treatment at the last follow-up. The mean BCVA improved from 0.81 ± 0.64 logMAR preoperatively to 0.41 ± 0.50 logMAR at the last follow-up (*P* < 0.001). BCVA improved in 46 patients (69.7%), was unchanged in 11 patients (16.7%), and was decreased in nine patients (13.6%); in 39 of 66 patients (59.1%), it was 20/40 or better at the last follow-up. After excluding patients who had undergone cataract surgery simultaneous with vitrectomy (31 patients) or during the postoperative period (10 patients), BCVA improved significantly from 0.67 ± 0.46 logMAR to 0.43 ± 0.46 logMAR (*P* = 0.044). Overall, the rate of IU recurrence in the postoperative period was lower in the 23- or 25-gauge vitrectomy group (10 of 36 patients, 27.8%) than in the 20-gauge vitrectomy group (12 of 30 patients, 40.0%), though the difference was not statistically significant (*P* = 0.310).

**Figure 1:**
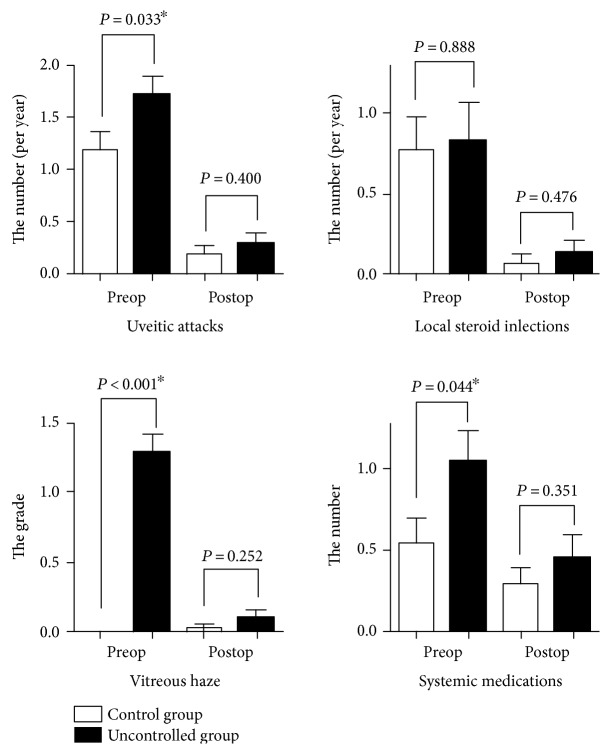
The average number of uveitis attacks, local steroid injections, the grade of vitreous haze, and systemic medications before and after vitrectomy were classified into the quiescent group and the active uveitis group based on whether there was complete inflammation control before surgery. There were no significant differences in the average number of uveitis attacks (0.19 versus 0.29, *P* = 0.400), local steroid injections (0.06 versus 0.13, *P* = 0.476), the grade of vitreous haze (0.03 versus 0.10, *P* = 0.252), and systemic medications (0.31 versus 0.47, *P* = 0.351) between the quiescent and active groups in the postoperative period, although there were significant differences in the average number of uveitis attacks (1.18 versus 1.72, *P* = 0.033), the grade of vitreous haze (0.00 versus 1.30, *P* = <0.001), and systemic medications (0.55 versus 1.07, *P* = 0.044) in the preoperative period. *P* value was calculated using an independent *t*-test. Asterisks indicate statistical significance between two groups.

The patients were also classified based on whether their intraocular inflammation was under complete control prior to surgery. The former group was defined as the “quiescent group” (group A, 36 patients) and the latter group was defined as the “active inflammation group” (group B, 30 patients). Between the two groups, there were no differences in age, gender, preoperative BCVA, or operative indications (Table 2). The period from the last preoperative uveitis attack to surgery was 16.81 ± 24.25 months in group A and 4.08 ± 3.08 months in group B (*P* = 0.012). The average numbers of uveitis attacks and systemic medications before surgery were higher in group B than in group A (Table 2, Figure 1). The patients in group B showed grade 1 vitreous haze in 23 patients (76.7%), grade 2 vitreous haze in 6 patients (20.0%), and grade 4 vitreous haze in 1 patient (3.3%). In all of the patients irrespective of the degree of inflammation control, the dosage of systemic steroids was increased in the perioperative period and tapered down slowly thereafter.

The average follow-up period after vitrectomy was similar between the two groups. The degree of intraocular inflammatory control and the BCVA improved significantly in each group (all *P* < 0.05). The proportion of patients with postoperative uveitis attacks (one or more events) did not differ statistically (group A, 30.6%; group B, 36.7%; *P* = 0.600). There was no significant difference in the degree of intraocular inflammatory control after vitrectomy (Table 3, Figure 1). BCVA at the last follow-up was also not significantly different (group A, 0.45 ± 0.57 logMAR; group B, 0.37 ± 0.41 logMAR; *P* = 0.516). However, the duration to the disappearance of postoperative inflammation was shorter in group A than in group B (*P* = 0.012) (Table 3).

Postoperative complications included secondary formation of an epiretinal membrane (4 patients, 6.1%), recurrent macular edema (3 patients, 4.6%), postoperative rhegmatogenous retinal detachment (3 patients, 4.6%), choroidal detachment (1 patient, 1.5%), and endophthalmitis (1 patient, 1.5%). Ten patients (15.2%) underwent cataract surgery due to cataract progression, and 7 patients (10.6%) required treatment for increased intraocular pressure. Complete control of intraocular inflammation before surgery did not influence the complication rate (*P* = 0.948).

Regardless of the preoperative inflammatory state, the degree of inflammation control in the preoperative period was strongly correlated with inflammation in the postoperative period by Pearson's correlation. The correlation coefficient (*r* value) of the average number of uveitis attacks between the preoperative and postoperative periods was 0.407, that of the average number of local steroid injections was 0.444, and that of the average number of systemic medications was 0.450 (all *P* < 0.01).

## 4. Discussion

We found that vitrectomy was helpful for relieving or stabilizing preoperative inflammation and achieving better long-term visual outcomes for IU patients with accompanying vitreoretinal complications. In addition, preoperative control of inflammation did not affect the long-term effectiveness of vitrectomy in IU patients.

Many authors have reported that vitrectomy resolved these complications, improved visual acuity, and alleviated intraocular inflammation in a variety of uveitis groups including IU [5–9, 15, 19–22]. Molina-Prat et al. [9] reported that visual acuity improvement was observed in 20 of 22 eyes (90.9%) and that the clinical course was improved in 19 eyes (86.4%), allowing for suspension of systemic treatment in 16 pars planitis patients. Our present results reconfirmed that vitrectomy was a useful treatment modality for IU: 44 of 66 patients (66.7%) suffered no uveitis attacks after vitrectomy and 46 of 66 patients (69.7%) showed BCVA improvement.

Complete preoperative intraocular inflammation control is generally considered to be mandatory for patients with a history of uveitis. Nussenblatt and Scott [10] recommended that intraocular inflammation be clinically quiescent at least three months before elective surgery is even considered. There is a strong consensus that complete control of intraocular inflammation should be achieved and maintained for three months prior to cataract surgery [11–14]. However, this protocol is not always used for vitrectomy. Several authors have reported improved inflammation control after vitrectomy, even though intraocular inflammation was not completely controlled preoperatively in different entities of uveitis [15–17]. Our results are in line with those of those previous reports: the complete control of intraocular inflammation did not influence the degree of postoperative intraocular inflammation. However, the time to the disappearance of postoperative inflammation was longer when intraocular inflammation was not controlled. All of these concordant results support the hypothesis that complete preoperative inflammation control is not an essential prerequisite in cases where vitrectomy is performed in IU patients. This can be explained by the fact that vitrectomy is a procedure whereby inflammatory mediators and cells (including immune complexes and cytokines) are removed from the vitreous, which is the source and site of inflammation in IU. Vitrectomy therefore can be considered a more fundamental treatment modality than medical treatment in inflammatory control. Kaplan [23] is credited with the concept that vitrectomy offers an alternative to systemic immunomodulatory drugs for uveitis control in some patients with IU. There has been only one small randomized pilot study by Quinones et al. [16], which reported that the resolution of IU was better achieved by vitrectomy than by immunomodulatory drugs in patients with IU who were refractory to steroid treatment. Kaplan [23] also indirectly supported our current results; however, a large, multicenter, randomized, prospective study would be needed to demonstrate whether or not vitrectomy is a valid first step prior to systemic therapy for the treatment of IU.

The disease duration in the active inflammation group B (26.93 months) was about 11 months shorter than that in the quiescent group A (37.43 months), and the last preoperative uveitis attack occurred about 17 months before surgery in group A, compared to four months in group B. This reflects the fact that group B was in the active phase of the natural course of IU, unlike group A, which was in the inactive phase; nevertheless, the decrease in postsurgery intraocular inflammation was similar in both groups. Our data suggests that early surgery can change the natural course of IU to a more rapid induction of the indolent phase, which also supports vitrectomy as a primary treatment of IU. Kroll et al. [24] reported that early vitrectomy in cases of juvenile IU often led to improved visual acuity and regression of inflammatory attacks, based on their experience with 25 patients.

Regardless of whether inflammation was controlled completely before vitrectomy, the overall degree of inflammation before vitrectomy was significantly correlated with the degree of inflammation in the postoperative period. Soheilian et al. reported that preoperative severity of uveitis was correlated with an improvement in inflammatory activity after vitrectomy [25]. This suggests that the degree of IU recurrence could be an intrinsic characteristic of individuals and that, accordingly, this might not change after surgery, even though vitrectomy significantly decreased the frequency of IU attacks. Conversely, this could mean that vitrectomy is needed earlier in patients with chronic and refractory IU.

The advent of small-gauge vitrectomy promises to increase the usefulness of vitrectomy in the treatment of IU. Our study did not show significant benefits to the recurrence rate after vitrectomy for IU due to our small sample sizes, but the small-gauge systems have advantages over conventional 20-gauge systems in terms of shorter operation time, faster healing time, and less postoperative discomfort [26]. Therefore, the invasiveness and trauma of vitrectomy should be diminished. In fact, employment of a small-gauge, cannulated vitrectomy system might also diminish secondary intraocular inflammation induced by the vitrectomy itself. Recent reports have suggested that microincision vitreous surgery can be a safe and efficacious approach for patients with uveitis [27, 28].

Our study does have some limitations. As a retrospective study, it has inherent limitations with respect to bias control. The patients did not receive their medical treatments according to a uniform protocol, and vitrectomy was performed by two surgeons. The number of patients with severe inflammation was relatively small in the active inflammation group because most patients were partially controlled by medications before surgery. Our study showed that vitrectomy is feasible without waiting until the inflammation is completely quiescent. We were able to determine conclusively, within the parameters of the study and on the basis of more than four years of postoperative follow-up, that vitrectomy was effective for IU patients in terms of inflammation control and BCVA improvement.

In conclusion, vitrectomy, which is usually indicated to resolve vitreoretinal complications, is effective in IU for control of intraocular inflammation and improved visual outcomes. This outcome may not be affected by whether the eye is completely quiescent or not before surgery. A randomized, prospective study will be necessary in order to determine if vitrectomy can be an effective alternative primary treatment modality for IU.

## Figures and Tables

**Table 1:** Demographic and preoperative data of patients diagnosed with intermediate uveitis.

Parameters	
Number	66 patients, 66 eyes
Age	48.76 ± 13.51 (min: 21, max: 75)
Gender (male : female)	40 : 26
Laterality (right : left)	39 : 27
Bilateral intermediate uveitis	13/66 (19.7%)
Preoperative best-corrected visual acuity (BCVA)^∗^	0.81 ± 0.64
Period from onset of uveitis to vitrectomy (months)	32.63 ± 40.95 (min: 3, max: 290)
Lens state	
Phakia/pseudophakia	59/7
Operation indications	
Epiretinal membrane	26/66 (39.4%)
Vitreous opacity	21/66 (31.8%)
Tractional retinal detachment	12/66 (18.2%)
Macular edema	5/66 (7.6%)
Vitreous hemorrhage	2/66 (3.0%)
Total	66
Postoperative follow-up period (months)	48.91 ± 40.38 (min: 12, max: 180)
BCVA^∗^ at final follow-up	0.41 ± 0.50

BCVA: best-corrected visual acuity; ^∗^BCVA was converted to the logarithm of the minimal angle of resolution.

**Table 2:** Comparison of demographic and preoperative data between the two groups classified according to complete control of intraocular inflammation before vitrectomy.

Complete control of inflammation before vitrectomy	Yes	No	*P* value
Number (patients)	36	30	
Age	47.50 ± 14.46	50.47 ± 11.79	0.371^∗^
Gender (male : female)	22 : 14	18 : 12	0.927^†^
Preoperative follow-up period (months)	37.43 ± 50.64	26.93 ± 23.33	0.315^∗^
Preoperative BCVA	0.73 ± 0.67	0.91 ± 0.61	0.287^∗^
The last preoperative uveitic attack before vitrectomy (months)	16.81 ± 24.25	4.08 ± 3.08	0.012^∗^
Operation indications			0.144^†^
Epiretinal membrane	15	11	
Vitreous opacity	8	13	
Tractional retinal detachment	10	2	
Macular edema	2	3	
Vitreous hemorrhage	1	1	
Total	36	30	
The average number of uveitic attacks in the preoperative period (per year)	1.18 ± 0.91	1.72 ± 0.79	0.033^∗^
The average number of local injections in the preoperative period (per year)	0.78 ± 0.99	0.82 ± 1.12	0.888^∗^
The grade of vitreous haze before surgery	0.00 ± 0.00	1.30 ± 0.65	<0.001^∗^
The average number of systemic medications before surgery	0.55 ± 0.94	1.07 ± 1.08	0.044^∗^

BCVA, best-corrected visual acuity (in logarithm of the minimal angle of resolution); *P* value was calculated using an independent *t*-test^∗^ or Pearson's chi-square test^†^.

**Table 3:** Comparison of the postoperative inflammation control and BCVA between the two groups classified based on complete control of intraocular inflammation before vitrectomy.

Complete control of inflammation before vitrectomy	Yes	No	*P* value
Number (patients)	36	30	
Postoperative follow-up period (months)	44.92 ± 36.41	54.37 ± 44.23	0.345^∗^
BCVA at final follow-up	0.45 ± 0.57	0.37 ± 0.41	0.516^∗^
Postoperative uveitic attack	11 (30.6%)	11 (36.7%)	0.600^†^
The average number of uveitic attacks in the postoperative period (per year)	0.19 ± 0.44	0.29 ± 0.52	0.400^∗^
The average number of local injections in the postoperative period (per year)	0.06 ± 0.34	0.13 ± 0.41	0.476^∗^
The grade of vitreous haze at final follow-up	0.03 ± 0.17	0.10 ± 0.31	0.252^∗^
The average number of systemic medications at final follow-up	0.31 ± 0.57	0.47 ± 0.77	0.351^∗^
The duration when postoperative inflammation disappeared (months)	1.74 ± 1.02	2.58 ± 1.47	0.012^∗^

BCVA: best-corrected visual acuity (in logarithm of the minimal angle of resolution); *P* value was calculated using an independent *t*-test^∗^ or Pearson's chi-square test^†^.
